# Supplementary material for: Impact of statin withdrawal on perceived and objective muscle function
Source: PLoS One. 2023 Jun 14;18(6):e0281178. doi: 10.1371/journal.pone.0281178 (PMC10266600; doi:10.1371/journal.pone.0281178)
Supplement: S1 Table — SAMS: statin-associated muscle symptoms; FFA: free fatty acids; FBGL: fasting blood glucose level; INS: insulin; TSH: thyroid-stimulating hormone; PTH: parathormone; eGFR: estimated glomerular filtration rate; CRP: C-reactive protein; RF: rheumatoid factor; Cr: creatinine; UR: urea; PA: pyruvic acid; LAC: lactic acid; LDH: lactate dehydrogenase; ALKP: alkaline phosphatase; Ca: calcium; Na: sodium; K: potassium; Mg: magnesium; NH4: ammonia; P: phosphorus; Cl: chloride; Data are expressed as mean ± standard deviation; Post value with * is statistically different from the pre value with p≤0.01; Post value with † is statistically different from the pre value with p<0.05. (DOCX) [file pone.0281178.s001.docx]

**S1 Table.** Pre- and post-statin withdrawal plasma variables

|  | SAMS  (n = 42 to 46) | | | | | | No SAMS  (n = 10 to 14) | | | | | | Controls  (n = 14 to 15) | | | | | | Time  p | ANOVA  Category  p | T*C  p |
| --- | --- | --- | --- | --- | --- | --- | --- | --- | --- | --- | --- | --- | --- | --- | --- | --- | --- | --- | --- | --- | --- |
|  | Pre | | | Post | | | Pre | | | Post | | | Pre | | | Post | | |  |  |  |
| FFA (mmol/L) | 0.47 | **±** | 0.17 | 0.48 | **±** | 0.18 | 0.40 | **±** | 0.13 | 0.51 | **±** | 0.22 | 0.53 | **±** | 0.28 | 0.45 | **±** | 0.24 | 0.64 | 0.82 | **<0.01** |
| FBGL (mmol/L) | 5.49 | **±** | 0.50 | 5.53 | **±** | 0.49 | 5.29 | **±** | 0.60 | 5.29 | **±** | 0.44 | 5.24 | **±** | 0.39 | 5.32 | **±** | 0.35 | 0.36 | 0.08 | 0.82 |
| INS (pmol/L) | 96.0 | **±** | 48.2 | 93.4 | **±** | 44.5 | 76.9 | **±** | 25.9 | 68.6 | **±** | 17.5 | 63.9 | **±** | 17.0 | 67.6 | **±** | 26.3 | 0.41 | **0.02** | 0.30 |
| TSH (mUI/L) | 2.50 | **±** | 1.32 | 2.67 | **±** | 1.34 | 1.93 | **±** | 0.85 | 2.31 | **±** | 0.96 | 1.99 | **±** | 0.64 | 1.79 | **±** | 0.69 | 0.22 | 0.08 | 0.09 |
| PTH (ng/L) | 43.0 | **±** | 13.8 | 44.7 | **±** | 12.4 | 43.4 | **±** | 18.0 | 47.1 | **±** | 23.6 | 49.9 | **±** | 13.7 | 47.9 | **±** | 16.7 | 0.48 | 0.47 | 0.41 |
| eGFR (mL/min/1.73m^2^) | 93.0 | **±** | 11.2 | 91.2 | **±** | 12.3 | 92.5 | **±** | 9.8 | 93.6 | **±** | 11.4 | 95.9 | **±** | 9.1 | 99.9 | **±** | 23.2 | 0.37 | 0.34 | 0.10 |
| CRP (mg/L) | 1.78 | **±** | 2.09 | 1.55 | **±** | 1.48 | 0.60 | **±** | 0.30 | 1.13 | **±** | 1.15 | 1.37 | **±** | 1.60 | 1.60 | **±** | 1.84 | 0.35 | 0.29 | 0.21 |
| RF (KUI/L) | 8.36 | **±** | 3.07 | 9.29 | **±** | 2.57^†^ | 9.08 | **±** | 2.47 | 10.4 | **±** | 2.5 | 9.27 | **±** | 2.52 | 9.47 | **±** | 2.00 | **<0.01** | 0.44 | 0.37 |
| Cr (µmol/L) | 76.0 | **±** | 11.9 | 77.3 | **±** | 13.8 | 77.1 | **±** | 14.5 | 76.4 | **±** | 15.0 | 79.1 | **±** | 10.6 | 79.5 | **±** | 12.1 | 0.67 | 0.56 | 0.43 |
| UR (mmol/L) | 5.57 | **±** | 1.02 | 5.61 | **±** | 1.05 | 5.30 | **±** | 1.55 | 5.45 | **±** | 1.43 | 5.52 | **±** | 1.51 | 5.95 | **±** | 1.29 | 0.10 | 0.68 | 0.34 |
| PA (µmol/L) | 74.3 | **±** | 24.9 | 76.6 | **±** | 27.3 | 57.7 | **±** | 15.4 | 58.8 | **±** | 13.9 | 66.6 | **±** | 23.5 | 61.5 | **±** | 23.5 | 0.99 | **0.01** | 0.53 |
| LAC (mmol/L) | 1.23 | **±** | 0.50 | 1.36 | **±** | 0.76 | 1.04 | **±** | 0.50 | 1.03 | **±** | 0.30 | 1.18 | **±** | 0.72 | 0.98 | **±** | 0.41 | 0.80 | 0.11 | 0.30 |
| LDH (U/L) | 170 | **±** | 22 | 163 | **±** | 21 | 154 | **±** | 26 | 161 | **±** | 33 | 166 | **±** | 17 | 164 | **±** | 23 | 0.79 | 0.49 | **0.02** |
| ALKP (U/L) | 68.8 | **±** | 20.1 | 64.8 | **±** | 17.6^*^ | 65.5 | **±** | 21.1 | 64.9 | **±** | 19.5 | 59.0 | **±** | 15.3 | 58.9 | **±** | 17.4 | 0.11 | 0.37 | 0.11 |
| Ca (mmol/L) | 2.35 | **±** | 0.06 | 2.34 | **±** | 0.08 | 2.39 | **±** | 0.09 | 2.37 | **±** | 0.07 | 2.35 | **±** | 0.10 | 2.36 | **±** | 0.06 | 0.45 | 0.34 | 0.72 |
| Na (mmol/L) | 141 | **±** | 2 | 141 | **±** | 2 | 141 | **±** | 1 | 140 | **±** | 2 | 141 | **±** | 2 | 141 | **±** | 2 | 0.37 | 0.67 | 0.09 |
| K (mmol/L) | 4.40 | **±** | 0.31 | 4.44 | **±** | 0.38 | 4.34 | **±** | 0.31 | 4.37 | **±** | 0.21 | 4.33 | **±** | 0.37 | 4.27 | **±** | 0.23 | 0.89 | 0.31 | 0.66 |
| Mg (mmol/L) | 0.86 | **±** | 0.06 | 0.86 | **±** | 0.05 | 0.86 | **±** | 0.06 | 0.86 | **±** | 0.07 | 0.85 | **±** | 0.07 | 0.84 | **±** | 0.05 | 0.28 | 0.59 | 0.95 |
| NH4 (µmol/L) | 22.5 | **±** | 10.7 | 24.0 | **±** | 8.3 | 18.9 | **±** | 8.0 | 22.1 | **±** | 10.4 | 24.0 | **±** | 9.5 | 23.9 | **±** | 9.4 | 0.31 | 0.65 | 0.81 |
| P (mmol/L) | 1.04 | **±** | 0.11 | 1.03 | **±** | 0.14 | 1.06 | **±** | 0.15 | 1.07 | **±** | 0.18 | 1.06 | **±** | 0.35 | 1.12 | **±** | 0.12 | 0.33 | 0.46 | 0.37 |
| Cl (mmol/L) | 101 | **±** | 2 | 102 | **±** | 3 | 102 | **±** | 2 | 102 | **±** | 2 | 102 | **±** | 3 | 102 | **±** | 3 | 0.69 | 0.83 | 0.41 |
