# Supplementary material for: Impact of statin withdrawal on perceived and objective muscle function
Source: PLoS One. 2023 Jun 14;18(6):e0281178. doi: 10.1371/journal.pone.0281178 (PMC10266600; doi:10.1371/journal.pone.0281178)
Supplement: S2 Table — SAMS: statin-associated muscle symptoms; * Framingham Score: values are means ± standard deviation and ANOVA p = 0.06. (DOCX) [file pone.0281178.s002.docx]

| Characteristics | SAMS | No SAMS | Controls |
| --- | --- | --- | --- |
| Type of statins | | | |
| Rosuvastatin, n (%) | 32 (52.5) | 6 (40.0) | - |
| Atorvastatin, n (%) | 18 (29.5) | 9 (60.0) | - |
| Fluvastatin, n (%) | 4 (6.56) | 0 (0.00) | - |
| Pravastatin, n (%) | 1 (1.64) | 0 (0.00) | - |
| Simvastatin, n (%) | 6 (9.84) | 0 (0.00) | - |
| Dose distribution per statin | | | |
| Rosuvastatin 2.5-10 mg, n (%) | 22 (36.1) | 4 (28.6) | - |
| Rosuvastatin 20-40 mg, n (%) | 10 (16.4) | 2 (14.3) | - |
| Atorvastatin 2.5-10 mg, n (%) | 12 (19.7) | 5 (35.7) | - |
| Atorvastatin 20-40 mg, n (%) | 6 (9.84) | 3 (21.4) | - |
| Simvastatin 2.5-10 mg, n (%) | 2 (3.28) | 0 (0.00) | - |
| Simvastatin 20-40 mg, n (%) | 4 (6.56) | 0 (0.00) | - |
| Fluvastatin 20-40 mg, n (%) | 4 (6.56) | 0 (0.00) | - |
| Pravastatin 20-40 mg, n (%) | 1 (1.64) | 0 (0.00) | - |
| Framingham Score*, % | 3.7 ± 3.5 | 2.3 ± 2.4 | 1.8 ± 2.1 |
| Duration of statin use before study enrolment | | | |
| <1-year, n (%) | 9 (19.6) | 0 (0.00) | - |
| >1-year, n (%) | 37 (80.4) | 14 (100) | - |
| Subject who has not tolerated a form of statin, n (%) | 15 (31.9) | 4 (26.7) | - |
| Form of statin not tolerated | | | |
| Atorvastatin, n (%) | 8 (53.3) | 3 (75.0) | - |
| Rosuvastatin, n (%) | 6 (40.0) | 1 (25.0) | - |
| Pravastatin, n (%) | 1 (6.67) | 0 (0.00) | - |
| Self-reported time of symptom apparition following statin reintroduction, weeks | 8.0 ± 18.3 | - | - |
| Family history (first degree) of cardiovascular disease, n (%) | 27 (47.4) | 8 (53.3) | 3 (20.0) |
| Family members (first degree) who took lipid-lowering drugs too, n (%) | 30 (73.2) | 12 (80.0) | 4 (25.0) |
| Family members who report side effects, n (%) | 6 (42.9) | 1 (10.0) | 0 (0.00) |
| Type of side effects reported by the family members | | | |
| Muscular | 3 (50.0) | 1 (100) | 0 (0.00) |
| Pain | 1 (16.7) | 0 (0.00) | 0 (0.00) |
| Muscle fatigue | 1 (16.7) | 0 (0.00) | 0 (0.00) |

**S2 Table.** Clinical profile of statin users
