# Supplementary material for: Impact of statin withdrawal on perceived and objective muscle function
Source: PLoS One. 2023 Jun 14;18(6):e0281178. doi: 10.1371/journal.pone.0281178 (PMC10266600; doi:10.1371/journal.pone.0281178)
Supplement: S1 Protocol — (PDF) [file pone.0281178.s005.pdf]

Muscle-Related Side Effects of Statins: Functional Impact, Mechanisms, and Potential Reversibility with Vitamin D Supplementation

1.0 PROJECT OVERVIEW

Amongst the most prescribed drugs worldwide, statins play a key role in health care by improving the cardiovascular outcomes of many patients. However, the use of statins is not without side effects. Besides the rare cases of rhabdomyolysis, other skeletal muscle-related side effects (MRSE) such as myalgia, cramps, and fatigue are estimated to affect 10% or more of the patient population under statin treatment [1]. Because approximately 10% of the population is taking statins on a daily basis [2], we can then extrapolate that over 300,000 Canadian patients are suffering from MRSE. Surprisingly little is known about the functional impact of these symptoms or the mechanisms responsible for their appearance. More importantly, health care professionals do not know how to deal with or relieve patients from MRSE. This unique project fills in this knowledge gap by combining functional, histological, and molecular approaches to improve our understanding of skeletal muscle-statin interactions and by testing an alternative treatment to reduce MRSE.

Objectives: The two main objectives of this study are the following:

1) *To test muscle strength and fatigability and investigate skeletal muscles at the histological and molecular levels in statin treated patients with and without MRSE; and*

2) *To assess the usefulness of vitamin D supplementation in improving muscle function and structure and reducing muscle-related side effects.*

More specifically, this project will enable us to determine (1) whether a loss of muscle strength and endurance can result from statin treatment, (2) whether statin therapy influences muscle morphology (in particular mitochondrial content and function) and increases muscle fiber degeneration in some susceptible subjects in relation to functional changes, and (3) if vitamin D supplementation can decrease statin-induced MRSE and muscle tissue damage while preserving muscle function and the cardiovascular benefits (decreased low-density lipoprotein cholesterol levels) of the drug.

Hypotheses: 1) Patients under statin monotherapy and suffering from statin-induced MRSE show reduced muscle strength and increased fatigability associated with an increased prevalence of cell damage, proteolytic markers, and perturbed mitochondria. 2) The cessation of statin monotherapy improves muscle function and reduces biological markers of muscle cell degeneration in patients with MRSE. 3) Vitamin D supplementation followed by statin reintroduction protects against statin-induced MRSE.

Patient population: Men and women aged between 30-60 years (inclusive) and affiliated with the cardiovascular prevention lipid clinic at the Laval University Hospital Research Centre (CHUL) will be included in this study. Patients treated with statin therapy for the first time in the last year with or without MRSE and with normal creatine kinase (CK) blood levels will be recruited. These patients must not have taken statins or lipid–lowering drugs before the last year or consume vitamin D supplements. MRSE must clearly have begun after statin introduction. These patients will be in low cardiovascular risk primary prevention, allowing us to modify their lipid-lowering drug regimen for a short period of time.

Impact: A better understanding of the cellular and molecular events leading to muscle pain, damage and dysfunction in the presence of statins is essential for the design of effective therapeutic modalities. We are convinced that this project will provide the basic foundation for this and may position Canada as a world leader on how to deal with MRSE associated with statins. Lastly, the present proposal also proposes an alternative treatment by testing the combination of statins and vitamin D supplementation to alleviate MRSE, an area in which clinical research and transferable solutions to patients are crucially needed.

2.0 BACKGROUND AND RATIONALE

HMG-CoA reductase inhibitors (statins) are effective and popular agents for reducing circulating LDL-cholesterol levels in both primary and secondary prevention of cardiovascular disease. In the last decade, there have been over 500 million prescriptions for statins dispensed in developed countries. The use of statins is expected to increase in Canada over the next few years, as the patent protection period for most major statins is expiring and cheaper generic versions come to market. For example, Pfizer's Lipitor (atorvastatin), the world's best selling medicine (sales nearing US$11.5bn globally in 2009, about US$1.2bn in Canada alone), will be facing competition from Apotex (Canada), Teva Pharmaceutical Industries (Israel), Watson Pharmaceuticals (US) and Ranbaxy Laboratories (India), who have all announced the launch of their atorvastatin products in Canada on the back of product approvals by Health Canada. Furthermore, the most recent Canadian guidelines [3] for the diagnosis and treatment of dyslipidemia and prevention of cardiovascular disease in the adult have broadened the inclusion criteria for pharmacological treatment as well as targeted even lower plasma LDL levels. Taken together, the patient population with prescribed statins in Canada is expected to increase significantly within the next few years and a better understanding of statin-induced MRSE is therefore very relevant for the Canadian population.

Statin-induced myopathy. Statins are generally well tolerated and very serious side effects appear to be rare [4]. The one notable serious adverse reaction to statin therapy is ***rhabdomyolysis*** (muscle symptoms with marked creatine kinase elevation and creatinine elevation [4]), which was observed early on with lovastatin in cardiac transplant patients [5]. In the worst cases, severe myoglobinuria accompanied by serum CK levels greater than 10 times normal, acute renal failure and even death can occur. Only a small fraction of patients who are treated with statins will develop such a severe myopathy (perhaps 1 in 10,000 patients) [6]. Based on FDA reports, the rate of fatal rhabdomyolysis was 16- to 80-fold greater for cerivastatin as compared to other statins, particularly at higher doses, but the mechanisms leading to such muscle toxicity are still poorly understood. In clinical practice, some non-specific predisposing factors have been pointed out and included advanced age, low body weight, female gender, use of multiple medications, multi-systemic disease, acute illnesses and major surgery [4]. Information on these issues as well as on the confusing area of low-grade myopathy (with normal CK) associated with statin use remains, however, extremely limited.

In addition to these rare but potential lethal cases of rhabdomyolysis, anecdotal and empirical observations indicate that other MRSE can result from statin use, including ***myalgia*** (muscle ache or weakness without elevated creatine kinase levels [4]), cramps, and reduced exercise endurance. These MRSE are generally believed to be more common than reported in statin clinical trials, in part because these studies were not designed to assess these particular symptoms. For example, a recent observational study of nearly 8,000 patients under high dose statins in primary care showed that 10% of patients presented MRSE, a prevalence that reached 16% for some statins [1]. These MRSE can be relatively severe and are an important quality of life issue, the pain often sufficient to alter the patients' normal physical activities. They can also lead to statin therapy cessation, preventing patients from achieving LDL cholesterol goals at any cardiovascular risk level. Furthermore, statin use may exacerbate muscle performance declines with impacts on health; *e.g.*, their use is associated with an increased risk of falls with aging without a concomitant decrease in muscle mass, an effect that may be reversible with statin withdrawal [7].

Although most studies have focused on middle-aged or older adults (typical of the statin-prescribed patient population), statin-related MRSE are not limited to older adults. In a study of young (24±6 yrs) professional athletes (15 men, 7 women) suffering from familial hypercholesterolemia, all ceased statin therapy due to myalgia. After withdrawal, all MRSE symptoms disappeared [8].

The impact of statin-induced MRSE on medication compliance is unknown. The persistence and adherence to medications that must be taken chronically are rather low. In fact, less than half of patients

prescribed chronic medications are compliant [9]. Statins are no exception to this observation. The proportion of patients in the province of Quebec who were 80% adherent over 2 years of statin treatment was less than 60% [10]. Of 100 patients starting statins for the primary prevention of coronary events, 44 will have discontinued therapy before any clinical benefit can be expected [11]. We suspect that statin-induced MRSE contribute significantly to this phenomenon, although this has not been studied.

Statin-induced MRSE remain an underappreciated phenomenon, and very little is known on their effects on patient compliance or on how to prevent or reduce these symptoms.

Statins, muscle strength, and exercise performance. Very few data are available on the effects of statins on muscle function. The authors of a recent review [12] examined published reports on the effects of statins on skeletal muscle strength and exercise performance and identified only 6 studies with data on the effects of statins on muscle strength, and 9 with data on exercise performance. Their main conclusions were that "there is insufficient data to determine if statins affect muscle strength and exercise performance" and that "there is suggestive evidence that these drugs may reduce muscle strength in older patients and alter energy metabolism during aerobic exercise". The paucity of data on these issues clearly warrants further studies. ***To our knowledge, no study has systematically examined the impact of statin-induced myalgia on muscle strength and endurance.***

Complicating matters further, a number of major confounders and technical problems are found in the few studies examining statin effects on muscle function. First, the characteristics of subjects under statins are often incomplete. For example, most of these studies do not distinguish between patients with or without statin-related myalgia. Also, in many of the studies, the precise methods used to assess muscle function are either poorly described, or are subject to significant error and are inappropriate for small group studies.

The first study to examine the possible associations of statins and muscle strength was that of Phillips *et al.* [13]. Hip flexion and abduction strength was measured in 4 patients with muscle myopathy, but with normal creatine kinase and statin blood levels. Compared to measures while off statins, statin use by these patients resulted in ~25% and ~19% decreases in hip flexion and abduction strength, respectively.

Using data from the Tasmanian Older Adults Cohort Study (TASOAC), Scott *et al.* [7] reported that statin users aged 50-79 years had significantly lower mean leg strength (isometric muscle strength of the quadriceps and hip extensors assessed with a dynamometer) that non-users. Over time, statin users also lost more strength and muscle mass compared to those who had stopped statin therapy after first entering the prospective study. These data suggest that statin use reversibly decreases muscle strength in older adults. In the observational Hertfordshire Cohort Study, no effect of statins on handgrip strength was noted in a group of men and women aged 59-73 [14]. In both of these studies no distinction was made between statin users with or without MRSE.

In terms of exercise capacity, data from published studies appear to be contradictory. A prospective study on the effects of 12 weeks of high-dose statin therapy (simvastatin 80 mg/day) in 10 patients aged 55-76 yrs failed to show any change in aerobic capacity, endurance, oxygen kinetics or muscle strength (chest press and leg press 1-RM) [15]. None of the subjects in this study reported symptoms of myalgia or cramps, leading the authors to conclude that in the absence of myalgia or myopathic symptoms, high-dose simvastatin treatment did not impair exercise capacity in hyperlipidemic older individuals.

In patients with stable congestive heart failure, atorvastatin treatment for 8 weeks improved flow-dependent brachial vasodilation and increased average 6 min walking distance by about 10% [16]. In patients with peripheral arterial disease, atorvastatin [17] and simvastatin [18] improved pain-free walk time when compared to placebo, presumably by improving the endothelial vasodilatory response to exercise. A recent meta-analysis [19] concludes that statins are the most efficient pharmacotherapy to improve mean walking distance in patients with intermittent claudication caused by peripheral vascular disease. Again, no information on MRSE status of the patients in these studies is available.

Eleven patients suffering from rhabdomyolysis or myositis (muscle symptoms with increased creatine kinase levels) were shown to have a reduced anaerobic threshold and tended to have reduced maximal oxygen consumption ($\dot{V}O_{2max}$) when compared to controls [20]. Whether such changes occur in patients suffering from statin-induced myalgia remains unknown.

These same patients suffering from rhabdomyolysis or myositis also demonstrated an increased respiratory exchange ratio during exercise [20]. A similar increase in respiratory exchange ratio during exercise was observed in type 2 diabetes patients under statin therapy [21]. These data suggest impaired fatty acid oxidation and an increased dependence on carbohydrate metabolism while under statin therapy, although this has not been evaluated in patients suffering from statin-induced myalgia.

Mechanisms of statin myotoxicity. Hypotheses attempting to explain statin effects on skeletal muscle cells generally relate to events leading to rhabdomyolysis and focus on reduced levels of products of the HMG-CoA reductase pathway. The most commonly cited are deficiencies in cholesterol (affecting membrane fluidity and function), coenzyme Q_{10} (resulting in perturbed mitochondrial respiration), and prenylated protein abnormalities (affecting intracellular protein messaging). Data supporting these hypotheses largely come from studies on cell culture or animal models of acute overdose [22]. Few data are available from human muscle biopsies, notably in relation to statin-induced myalgia.

Evidence from a small group of patients suffering from statin-induced rhabdomyolysis support effects of statins on skeletal muscle through prenylated protein anomalies. Phillips *et al.* [23] showed a tendency for elevated mRNA levels of the muscle atrophy signal atrogin-1 (which can be induced by reduced prenylation of G-proteins) and significantly decreased protein levels of the prenylated protein Ras. Although a number of histological and ultrastructural markers were also shown to be present in patients suffering from rhabdomyolysis, these were no more frequent than in age-matched controls. No differences in mitochondrial content, enzyme activities, muscle coenzyme Q_{10} concentrations or cholesterol levels were seen between groups. Limitations of this study include the absence of data on any changes to these parameters after statin withdrawal. Also, the inclusion of subjects from an age range reaching well into the late 80's (mean 64 yrs, range 49-87) is a potential confounder, since an acceleration of the loss of muscle function (*e.g.*, strength) is observed around 60 years of age (discussed in[24]).

The only data we are aware of that examined histological changes in muscle biopsies from patients with statin-related myalgia are from Phillips *et al.* [13]. In a very small group of only 4 patients, evidence of myocyte damage or mitochondrial dysfunction was observed that included abnormally increased lipid stores, fibers that did not stain for cytochrome oxidase activity, and ragged red fibers. These observations were reversible in 3 of the 4 patients following statin withdrawal within 2 months.

These data support the hypothesis that changes at the level of the skeletal muscle cell, in particular in terms of mitochondrial structure and metabolic function, could be associated with statin-induced MRSE. Whether they precede or result from statin use remains unclear.

Vitamin D deficiency and myalgia. Vitamin D deficiency is highly prevalent worldwide and low serum levels of 25-hydroxyvitamin D (25(OH)D) are associated with higher risk of cardiovascular morbidity and mortality [25]. Vitamin D deficiency is also associated with a number of muscle symptoms, *e.g.*, myalgia strikingly similar to that resulting from statins [26], and has also been associated with myositis [27]. Vitamin D insufficiency is highly prevalent in patients with type 2 diabetes and may cause neuropathic pain in the inadequate (serum 25(OH)D levels of 40–60 nmol/l) rather than severely deficient range (25(OH)D <30 nmol/l) [28].

Vitamin D and muscle morphology and performance. Effects of vitamin D levels on muscle performance have been reported in a few studies, vitamin D possibly improving muscle strength and function through a highly specific nuclear receptor in muscle tissue. Studies showing associations between vitamin D status and physical performance walking speed, sit-to-stand times, handgrip strength,

and risk of falls are mostly observational and in older adults (reviewed in [29-33]). At the muscle cell level, vitamin D deficiency is associated with predominantly type II muscle fiber atrophy (more so in severely deficient patients), enlarged interfibrillar spaces and fat infiltration, fibrosis and glycogen granule accumulation [34].

Whether vitamin D supplementation can itself impact muscle strength or endurance or correct modifications observed with deficiency remains a matter of significant controversy. Studies into this area have mostly been performed in elderly patients, in the context of age-related sarcopenia or functional loss, and in the vast majority of cases with concurrent calcium supplementation. A number of studies have reported positive functional impacts of vitamin D supplementation on physical performance and risk of falls in the elderly, as reviewed in [29].

Only two studies have to our knowledge examined the effects of vitamin D supplementation on muscle morphology, both in patients with low vitamin D status. In a small, uncontrolled study, *vastus lateralis* biopsies before and after 3-6 months of 1-alpha-hydroxyvitamin D and calcium supplementation were recovered from elderly women suffering from bone loss [35]. Increased succinate dehydrogenase activity, phosphagen levels, as well as an increase in type IIA fiber proportion (at the expense of IIX fibers) and cross-sectional area were noted after the supplementation period. A randomized controlled trial in elderly women with post-stroke hemiplegia (48 supplemented, 48 placebo) taking 1000IU/day of vitamin D_2 over 2 years resulted in increased type II fiber diameter and proportion as well as improved muscle strength in the vitamin D-treated group [36]. Positive correlations between type II fiber area and serum 25(OH)D levels were noted before and after treatment in this study. Increased type II fiber area is proposed to account at least in part for increased strength in these studies. However, whether these changes to fiber morphology resulted from direct effects of vitamin D on muscle cells remains unknown, as do the effects of vitamin D supplementation in patients with adequate or normal vitamin D status.

Although these data suggest that vitamin D supplementation could lead to skeletal muscle morphology or functional changes, these changes are modest and would only be beneficial to patients suffering from statin-induced MRSE.

Vitamin D and statins. The relationship between statins and vitamin D metabolism is not well-understood, although 7-dehydrocholesterol is the synthetic precursor of both cholesterol and vitamin D. Whether vitamin D insufficiency potentiates statin-induced myalgia, and whether statins contribute to vitamin D deficiency (in the circulation or at the tissue levels), is still poorly documented. The latter seems unlikely as atorvastatin and rosuvastatin treatments have been shown to increase serum 25(OH)D and active hormone 1,25-dihydroxyvitamin D concentrations [37, 38]. Wu-Wong *et al.* [39] showed that statins do not directly activate the vitamin D receptor *in vitro*, although it still remains to be determined whether some metabolites of statins could.

Vitamin D deficiency could itself impact statin metabolism. Simvastatin, lovastatin and atorvastatin are substrates of CYP3A4, the latter also displaying 25-hydroxylase activity *in vitro* [40]. In addition, CYP3A4 expression is also regulated by the vitamin D receptor pathway in at least some human tissues [41]. Vitamin D deficient states could thus lead to preferential shunting of CYP3A4 for hydroxylation of vitamin D, resulting in reduced availability of CYP3A4 for statin metabolism, contributing to statin-induced toxicity by increasing circulating drug concentrations. In agreement with this hypothesis, vitamin D supplementation (800 IU/day for 6 weeks) increased vitamin D-25-OH metabolites while reducing atorvastatin active metabolite concentrations in a trial including 16 men and women [42].

Clearly a complex interplay between vitamin D and statins occurs. Further studies are required to clarify the relationship between vitamin D deficiency and statin-induced myopathy and to confirm whether vitamin D repletion might resolve myalgic symptoms and allow statin reintroduction in a wide range of patients.

Early evidence does support a role for vitamin D supplementation in reducing statin-induced MRSE. Recent data from non-controlled trials show reversible myositis-myalgia in statin-treated patients receiving vitamin D repletion [43, 44]. In the study of Ahmed *et al.* [43], a greater proportion of statin-using patients with myositis-myalgia (64%) were found to have low levels of 25(OH)D when compared to asymptomatic statin users (43%). In this study, 38 vitamin D deficient myalgic statin-taking patients were supplemented with vitamin D (50,000IU/week for 12 weeks), resulting in increased serum vitamin D levels as well as resolution of myalgia in 92% of the cases. In the study of Lee *et al.* [44], 6 vitamin D deficient patients who had developed myopathy following statin introduction agreed to be rechallenged with statin therapy (same statin) following vitamin D supplementation, consisting of oral vitamin D_3 does of 1,000 – 10,000 units/day. Following repletion to reach normal 25(OH)D levels (84.5 ± 7.5 nmol/l), in four of these patients statin reintroduction was tolerated for at least 6 months without recurrence of myalgia. Notably, statin rechallenge was more likely to be successful in patients who achieved the highest post-treatment 25(OH)D levels (>80 nmol/l). ***This suggests that achieving higher levels of serum 25(OH)D, even well above the currently proposed desirable level threshold of 75 nmol/l, might be beneficial in protecting against statin-induced myalgia***.

According to recent data from the Canadian Health Measures Survey of 2007-2009 and as analyzed by Statistics Canada [45], well over half (~55-65%) of Canadian adults (men and women) aged 40-79 years did not reach desirable 25(OH)D levels of 75 nmol/l. ***This would suggest that a large majority of Canadians under statin treatment has vitamin D metabolite levels well below those proposed by Lee et al. [44] and that vitamin D supplementation may help to alleviate drug-induced myalgia.***

3.0 STUDY DESIGN AND EXPECTED FINDINGS

A flow diagram summarizing the study design and an outline of patient participation can be found in **Figure 1** and **Table 1**.

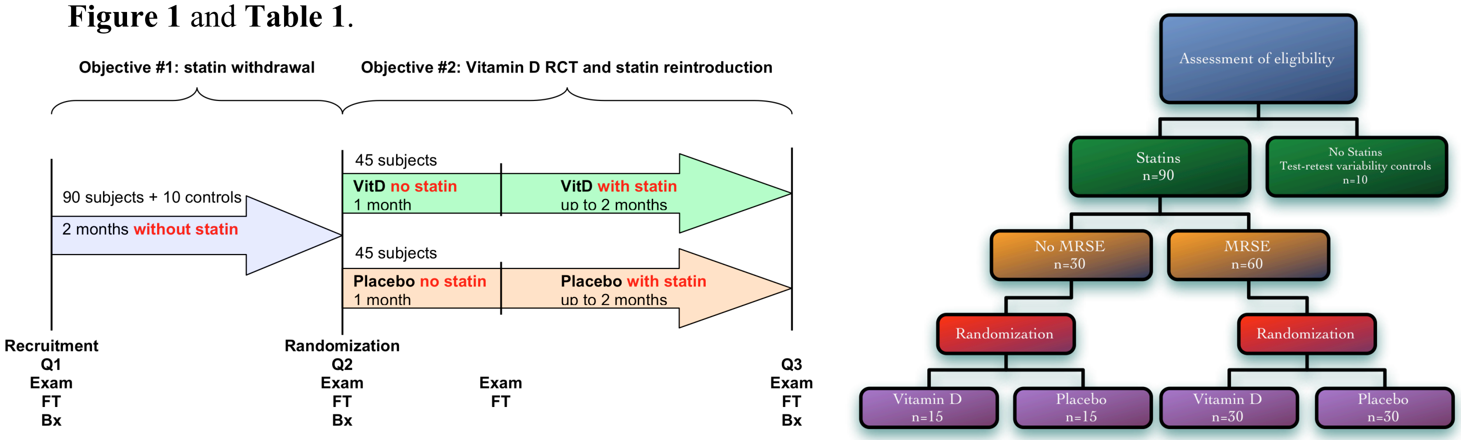

Figure 1: Simplified timeline and flow diagram of proposed study. Q1, Q2, and Q3: questionnaires; Exam: physical examination (including anthropometry) and blood work; FT: functional muscle testing; Bx: *vastus lateralis* muscle biopsies (in a subset of patients).

Table 1: Subject population breakdown in proposed study. Subjects from objective #1 will be randomized into experimental (vitamin D) and control (placebo) groups for objective #2 based on age, sex, MRSE and 25(OH)D levels.

**Group**	**Objective #1**	**Objective #2**
Statin taking with MRSE	60 (20 with biopsies)	30 vitamin D (10 with biopsies)<br>30 placebo (10 with biopsies)
Statin taking without MRSE	30 (10 with biopsies)	15 vitamin D (5 with biopsies)<br>15 placebo (5 with biopsies)
Controls (no statins)	10 (5 with biopsies)	—

Inclusion criteria: Healthy and sedentary or moderately physical active (less than 1 hour of leisure-time physical activity per week) men and women aged 30 to 60 years (inclusive). Patients placed on statin therapy for the first time in the last year with or without MRSE and with normal CK blood levels.

Exclusion Criteria: Previous use of statins (before the last year); Current treatment with other lipid-lowering drugs; Natural medicine affecting lipid metabolism; Creatine kinase (CK) levels above the normal range (>180 U/L for women; >250 U/L for men); Clinical vitamin D deficiency (25(OH)D levels below 12.5 nmol/L); Impaired liver or kidney function; Untreated hypo- or hyperthyroidism; Treatment with other medications known to increase risk of myopathy in statin-treated patients (cyclosporine, anti-protease, azole antifungals…); Existing infection requiring antibiotic therapy; Consumption of greater that 60 ml of grapefruit juice per day; Inherited muscle disorders or myopathy; Polymyositis or inflammatory myopathy; Use of corticosteroids; Comorbidities resulting in muscle or bone pain (fibromyalgia, arthritis, sensory or intrinsic neuropathy, vertebral disease, loss of reflexes, muscle group atrophy); History of elevated CK (known or unknown etiology); Unexplained cramps; Known sickle cell trait; Cancer within the 5 years prior to study entry; Diabetes; Stroke, coronary artery or peripheral vascular disease; Physical disability or previous injury interfering with exercise testing; Pregnant or breastfeeding; Depression (in last 3 years) or treatment with antidepressants; Use of anti-psychotic drugs; Alcohol abuse.

Power calculations. Group sizes are derived from power calculations for changes in objective measures of muscle function as described in **Appendix 2**.

OBJECTIVE #1: To test muscle strength and fatigability and investigate skeletal muscles at the histological and molecular levels in statin treated patients with and without MRSE

Subjects. Men and women aged between 30-60 years (inclusive) and affiliated with the cardiovascular prevention lipid clinic at the CHUL will be included in this study. Only patients placed on statin therapy for the first time in the last year with or without MRSE (self-reported and assessed by questionnaires) and with normal CK blood levels will be recruited. These patients must never have taken statins or lipid–lowering drugs before the last year or consume vitamin D supplements, and their MRSE must clearly have begun after statin introduction. These patients will be in low cardiovascular risk primary prevention, allowing us to modify their lipid-lowering drug regimen for a short period of time. Informed written consent, to be approved by the CHUL ethics committee, will be obtained from each subject before entering the study. The lipid clinic at the CHUL is one of the largest in Canada, over 6000 patients seen or followed each year. The vast majority (estimated at 85-90%) of these patients are prescribed statins, and based on the personal experience of doctors at the clinic roughly 10% of these present symptoms of MRSE. A survey of patient records at the CHUL lipid clinic from June 2010 to February 2011 reveal nearly 300 patients with MRSE in our age range. Thus, we do not anticipate any need to recruit from other centers or through the media to reach our subject number goals, but these options remain available to us should they be required.

An extended screening profile for exclusion of inflammatory and metabolic myopathies will be performed. It is important to note that initial 25(OH)D levels will not be used as a criterion for subject recruitment (except for clinical vitamin D deficiency defined in our exclusion criteria). Vitamin D status (serum 25(OH)D levels), in addition to supplementation itself (in objective #2), will instead be assessed as potential contributors to improve statin tolerance in post-study statistical analyses.

One hundred (100) patients will be recruited for objective #1 (60 with MRSE, 30 without MRSE and 10 controls) for muscle function testing and a subset of 35 of them will also be investigated by percutaneous muscle biopsies. Ninety patients will be randomly distributed for objective #2 (60 with

MRSE, 30 without MRSE) for muscle strength and fatigability testing, and 30 of them will also be investigated by muscle biopsies (see figure 1 and table 1 for details).

Functional assessment and blood samples

A case-control approach will be used to compare patients under statin treatment with and without MRSE. After initial testing while under statin treatment, statin treatment will be stopped for 2 months followed by retesting. Recent studies have shown that most statin MRSE as well as structural muscle damage appearance/disappearance occurs within a two month period of statin introduction/withdrawal [1, 13]. A control group of 10 statin-naïve patients will also be included for test-retest variability assessment of all parameters.

Following screening, 60 patients presenting MRSE (assessed by a standardized questionnaire; see **Appendix 3, questionnaire 3**) under statin monotherapy (any statin, any dosage) as well as 30 patients without MRSE under statin monotherapy (paired for statin and dosage with the MRSE cohort) will be evaluated. Muscle strength and fatigability will be tested by standardized procedures before and after two months of stopping statin monotherapy. Briefly, strength of the extensors and flexors muscles of the dominant leg will be quantified with a Biodex isokinetic dynamometer available in our research centre. Forces will be tested during 3 reps at 60 and 180 deg/sec and endurance during a 15 reps test at 180 deg/sec. These tests will be preceded by a 5 reps warm-up at 60 deg/sec including one movement at maximal voluntary contraction [46]. Each maximal concentric contraction will be performed in the first 90 degrees of the knee range of motion. Patients will sit in an upright position and mechanical axis and resistance pads will be aligned according to the manufacturer's guidelines. The results will be reported in Newton-meters (Nm). Peak torque, rate of torque development and total work values will be obtained following these isometric and isokinetic measurements. These groups of muscles were selected because muscle weakness or myalgias with statins are most frequently localized in lower limbs. Handgrip strength of the dominant hand will also be tested in a static mode with a Jamar dynamometer (best of 3 repetitions). These testing procedures are well standardized, easy to perform and very reproducible, and are routinely used in the department of physical therapy at our centre Test-retest variability for the Biodex measurements is shown to be within ±15% [46], which our personal experience confirms. The primary efficacy parameter will be the percent change for these measurements from baseline to end of each phase of the study.

Before each muscle function test, venous punctures will also be collected to assess muscle and liver enzyme contents as well as 25(OH)D concentration in serum (DiaSorin RIA). These serum assays are routinely performed at the clinic and research center of CHUL. The percent change in serum CK, ALT, AST and creatinine from baseline to end of each phase of study will be evaluated for each patient. The vitamin D status of patients will be assessed using serum 25(OH)D levels. Plasma (or serum) 25(OH)D concentration is generally considered to be the best metabolite to reflect vitamin D status [47]. Patients with abnormal clinical results will receive standard medical care for their condition.

Preliminary data. To demonstrate the feasibility of our approach for evaluating functional changes upon drug withdrawal in patients suffering from statin-related MRSE, preliminary data have been gathered on muscle strength and endurance in 9 patients and are presented in **APPENDIX 1**. Part of this preliminary data has been presented in abstract form [48].

Six men and 3 women (57 ± 9 years) with MRSE that could affect their compliance to statins were subjected to a preliminary assessment of the functional capacity of knee flexor and extensor muscles when taking statins, and to a second evaluation 2 to 3 months after drug cessation. The strength of extensors and flexors was measured in a sitting position on a Biodex isokinetic dynamometer after 3 maximal contractions at 60 degrees per second. Power, endurance and fatigability were measured after 15 repetitions at 180 degrees/second. Muscle symptoms while taking statins and after cessation were documented as well as blood CK and AST levels.

Subjects (see **APPENDIX 1, Table 1**) in our study were statin users and had muscle side effects (cramps, myalgia, reduced muscle endurance) evaluated with our in-house questionnaire (see **APPENDIX 3, questionnaire 3**). All subjects were in primary cardiovascular prevention. Different statins were used by different participants, the majority at low doses (the highest being 20 mg).

After statin withdrawal, all subjects reported important decreases in the level of pain experienced (**APPENDIX 1, Figure 1**), and 70% showed functional improvements in at least one Biodex measure of muscle performance (**APPENDIX 1, Figure 2**). On average, the strength of the extensor and flexor muscles increased by 22±43% and 17±28% respectively, power by 28±64% and 29±41%, and endurance by 28±57% and 20±35% (means±SD). No effects were observed on blood CK and AST values (**APPENDIX 1, Table 2**).

Our preliminary results show that cessation of statin therapy increased muscle functional capacity in the majority of subjects who experienced *a priori* muscle discomfort. ***Since this condition could affect the pharmacological compliance and potentially modify the cardiovascular benefits of statins, it is imperative to better characterize this dysfunction and develop approaches to reduce its impact***.

Histological assessment of skeletal muscle

For a subset of 30 of the patients in the study (20 presenting MRSE and 10 without MRSE), percutaneous needle biopsies of the *vastus lateralis* muscle will be performed, before and after two months of discontinuation of statin monotherapy, in our outpatient surgical center. Dr. Jean Doré (Laval University), who has performed this procedure literally hundreds of times over the years, will be responsible for biopsies (**see letter of collaboration**). This technique is relatively painless (especially in patients with normal CK), well tolerated, can be performed within 20 minutes, and is routinely used in our laboratory for repeated measures over time [49]. A control group of 10 statin-naïve patients will have the same muscle functional testing and venous punctures collection at the start of the study and two months later; 5 of these will also have muscle biopsies.

Muscle biopsies will be analysed histologically with standard muscle stains and also frozen for future analyses using methods routinely used in our laboratory (*e.g.*, [49] and **APPENDIX 4, publications 2, 3 and 4**). Mitochondrial-like myopathy features associated with statin-myopathy (lipid droplet accumulation in type 1 fibers by oil red O staining, ragged red fibers, cytochrome oxidase-negative myofibers, mitochondrial protein profiling), myocyte fiber morphometry (size, type) and degenerating/regenerating fiber distribution (indicated by central nuclei stained with hematoxylin-eosin) will be assessed ([50, 51], and **APPENDIX 4, publications 2 and 3**). Enzyme activities and protein levels relating to energy metabolism (citrate synthase, cytochrome-c oxidase, hydroxyacyl-CoA dehydrogenase) or proteolysis (atrogin, Murf) will also be measured (see [52] and **APPENDIX 4, publications 2 and 3**). Mitochondrial protein expression profile for PPAR-γ coactivator-1α (PGC-1α) and downstream target proteins, protein import machinery components (Hsp60, Tom40 and Tom22), fusion proteins (mitofusin 2 (Mfn2) and optic atrophy 1 (Opa1)), fission protein (Drp1), apoptosis related proteins (Bax and Bcl-2), and autophagy proteins (Beclin 1 and light chain 3 (LC3-II)) will be assessed using previously established Western blotting methods (**APPENDIX 4, publication 4**) in collaboration with Dr. David Hood (York University; **see letter of collaboration**) The secondary efficacy parameters will be the qualitative or quantitative changes observed from baseline to end of each phase of the study.

<u>EXPECTED FINDINGS AND OUTCOMES</u>. ***This first phase of the proposal will allow us to confirm the loss of muscle functional capacity, as well as the nature of skeletal muscle structural and biochemical changes and tissue damage in those patients presenting MRSE***.

Primary outcomes for this objective include: (1) the demonstration of myalgia resolving with statin cessation and (2) the demonstration of reduced muscle function (strength and endurance) in

patients suffering from statin-related myalgia; a secondary outcome will be (3) the assessment of muscle histological, ultrastructural or metabolic changes potentially relating to expression of MRSE (in particular, muscle mitochondrial rearrangements are predicted).

OBJECTIVE #2: *To assess the usefulness of vitamin D supplementation in improving muscle function and structure and reducing muscle-related side effects.*

Trial design

This study will use an outpatient, randomized, double-blind (participants, research nurse, coordinator, investigators, interviewers, statisticians, treating physicians), prospective trial design with stratification on MRSE status, plasma 25(OH)D status, age and sex, with repeated measures at 4 weeks post-randomization and at up to 8 weeks post-statin reintroduction. This is diagrammed in Figure 1. Participants (from Objective #1) will first be divided into 2 groups according to their MRSE status: statin taking with MRSE and statin taking without MRSE. Participants in each group will then be randomly assigned to either the experimental (vitamin D) or the control (placebo) arm. The placebo will as closely as possible match the color, taste and consistency to the vitamin D administered in the experimental group.

The research coordinator and other members of the research team will be responsible for recruitment, testing, data collection and follow-up. Allocation of participants to treatment arms and statin, vitamin D and placebo supply and distribution will be under the responsibility of the Department of Pharmacy of the CHUL (see budget supporting document).

Vitamin D status will not be used to select subjects for initial (Objective #1) enrolment in the study, but will be used to stratify subjects in the RCT. Subject vitamin D status will be defined using cut-offs discussed in a recent Statistics Canada Health Report [45].

In the experimental group (n=45; 30 with MRSE under statin monotherapy, 15 without MRSE under statin monotherapy from objective #1), statins will be reintroduced following one month of oral vitamin D supplementation (Vitamin D_3 [cholecalciferol], Laboratoire Riva Inc., Blainville, Québec; 4 x 10,000 IU/week, 40,000 IU/week total). This falls within the range of average daily dosage used in the studies of Lee *et al.* [44] and Ahmed *et al.* [43] that showed relief from myalgia and allowed statin reintroduction in patients with low serum 25(OH)D levels. This also falls within the generally acceptable upper limit of daily vitamin D intake, the "no observed adverse event level" being 10,000 IU per day, and well below the "lowest observed adverse event level" of 40,000 IU per day (as discussed in [45]). Patients will be rechallenged with the same statin and at the same dose they were taking at the onset of objective #1. Patients will continue to supplement with vitamin D during the statin rechallenge period, which will last for a maximum of 2 months. The patients may request at any time over this period the cessation of statin therapy if they consider any reappearance of MRSE to be too severe to continue. Based on the clinical experience of Dr. Bergeron (co-PI), patients rarely develop muscle symptoms in less than 2 weeks following statin introduction.

In the control group (n=45; 30 with MRSE under statin monotherapy, 15 without MRSE from objective #1), no vitamin D supplementation will take place prior to statin reintroduction, subjects receiving instead a placebo provided by the pharmacy service of the CHUL. Placebo supplementation will also continue following statin reintroduction (1 month after the start of the RCT). Readers are referred to figure 1 and table 1 for more details on patient assignment.

Participants will be asked by the study coordinator, by phone, a few days before their visits at 4 weeks after randomization and at end-of-trial, to bring their bottles of capsules for their visit to the CHUL. The pharmacy will then check their apparent compliance by counting the remaining capsules and surveying the participants on their supplementation practices. A compliance rate of at least 80% will be required for successful outcome in the trial.

The changes in 25-OH vitamin D, calcium, phosphate, alkaline phosphatase, and parathyroid hormone, from baseline to end of each phase of study will be studied in all patients to evaluate the

impact of stopping statin monotherapy and the rechallenge of statin monotherapy under vitamin D repletion. As described in objective #1, and for both groups, muscle strength and fatigability tests and venous punctures (particularly for 25-OH vitamin D level assessments) will be obtained immediately prior to statin reintroduction (after one month of vitamin D or placebo). These and muscle biopsies will also be taken at the end of the statin-reintroduction period (within 1 week of the maximum 2 months trial length or from when patients cease statin use due to reappearance of important MRSE).

Muscle biopsies of the *vastus lateralis* will be obtained for 30 of these patients: 10 presenting MRSE and receiving vitamin D, 10 presenting MRSE and not receiving vitamin D, 5 without MRSE and receiving vitamin D, and 5 without MRSE and not receiving vitamin D. These will be the same patients that had biopsies in objective #1, allowing us to evaluate changes to skeletal muscle tissue over time. Our laboratory has extensive experience in multiple biopsies from the same patient over time, and we have published numerous papers using this approach (*e.g.*, [49, 53-57]).

In addition to the testing and questionnaires outlined in Figure 1, phone interviews will be performed every 2 weeks during the RCT to assess adverse events. An adverse event is any untoward medical occurrence in a study subject that is temporally associated with the use of a medicinal product, regardless of its potential relationship to the medicinal product. An adverse event, therefore, can be any unfavorable or unintended sign, symptom or disease (new or exacerbated), whether or not related to the study drug. The severity of an adverse event will be scored according to the following scale:

Mild: Awareness of sign or symptom, but easily tolerated
Moderate: Discomfort enough to cause interference with usual activity
Severe: Incapacitating with inability to perform usual activities

Participants will be provided with the adverse event guide presented in **Appendix 3** to help classify the nature and intensity of adverse events during phone interviews and in questionnaires.

Participants will be required to provide informed consent, will be assured confidentiality and will be offered the possibility to withdraw from the study at any time and without justification. Premature withdrawals and their causes will be carefully documented. Subjects who withdraw before the required sample size has been attained will be replaced; those who withdraw after the required sample size has been attained will not be replaced.

EXPECTED FINDINGS AND OUTCOMES. ***This second phase of the proposal will allow us to confirm that MRSE suffering patients may be protected against statin-induced myalgia as well as for muscle function and biological markers of muscle cell degeneration after vitamin D supplementation. In addition, these data will demonstrate a significantly improved capacity to achieve lower LDL-cholesterol levels in cardiovascular prevention while reducing the risk of muscle side-effects from statin use.***

Primary outcomes for this objective include: (1) reduced functional impact of statins on muscle strength and endurance in the vitamin D supplemented group, and (2) a greater tolerance of statin reintroduction (*i.e.*, fewer self-reported MRSE) in the group receiving vitamin D supplementation; secondary outcomes will be (3) the normalization of muscle histological, ultrastructural, and mitochondrial (metabolic) abnormalities with vitamin D supplementation and (4) the demonstration of improved statin tolerance with vitamin D supplementation regardless of initial 25(OH)D status.

4.0 Clinical follow-up and safety concerns

Clinical and anthropometric measures. In addition to medical history, traditional blood work (glycemia, insulinemia, lipid-lipoprotein profiles...) as well as anthropometric measurements (body weight, height, waist circumference...) will be obtained for all patients over the course of the study. Other specific analyses are presented in the appropriate sections that follow.

Statin efficacy. The percent change in serum total cholesterol, LDL-cholesterol, apolipoprotein B, HDL-cholesterol, apolipoprotein A-1 and triglycerides, from baseline to end of each phase of study will be studied in all patients to evaluate the impact of discontinuation of statin therapy and the rechallenge of statin monotherapy under vitamin D repletion on cardiovascular risk factors.

Questionnaires (see APPENDIX 3 for all questionnaires). The PIQ6 Pain impact questionnaire will be used to assess the perceived impact of MRSE on everyday activities. Perceived general health will be assessed using the SF-12v2 Health Survey Scoring questionnaire. An in house questionnaire will be used to evaluate the nature and perceived intensity of MRSE. This questionnaire will also explore aspects of medical history (such as recent illness, other potentially related diseases, family history of pain) that could impact MRSE. Three versions of this questionnaire will be used: one to evaluate subjects in the screening and pre-withdrawal phase, a second following 2 months of statin withdrawal, and a third at the end of the statin reintroduction period in the vitamin D randomized placebo controlled trial.

Adverse events and other serious events. Participants will immediately be withdrawn from the trial should any change in their health occur over the course of the study that warrants immediate medical care using standard medical guidelines.

5.0 STATISTICAL ANALYSES

Although there are no published data on the loss of muscle force in humans following statin treatment, experiments using men with coronary artery disease showed that n = 10 to 20 is sufficient to distinguish differences between groups [58]. Based on the high accuracy of the Biodex apparatus and our preliminary data, we calculate that a total of 60 MRSE suffering patients will be sufficient to demonstrate effects on muscle function by statin withdrawal or vitamin D supplementation (30 per group, one with vitamin D, the other with placebo - see **APPENDIX 3** for Power Calculations). These sample sizes are similar to those producing data showing reversible myositis-myalgia in statin-treated low serum 25(OH)D patients receiving vitamin D repletion [43, 44]. For biopsies, the number of subjects in each study group was determined based on our previous work (*e.g.*, [49]), biopsy feasibility and the availability of patients from our clinic [59]. From the patient case reports documented with some of these MRSE and considering the analytical precision of the methods proposed, we are confident that we will show statistically significant differences between MRSE suffering and not suffering patients [13].

Statistical analyses will test the comparison of subgroups (with/without symptoms and with/without vitamin D repletion) on the percent change from baseline (end – baseline) of specific measurements. Changes from baseline (end – baseline) of all clinical muscle symptoms will be also analyzed. Normal distribution of fasting marker levels will be examined and, if necessary, they will be normalized by transformation prior to statistical analyses in order to reduce the skewness of their distribution. One-way ANOVA will be used to compare means of continuous variables. Differences in proportions (e.g. proportion of patients having increments in one or more specific markers) will be compared using either the Chi-square test, or the Fisher's exact test when appropriate. Statistical analyses will be performed using SAS (SAS Institute Inc.) and/or JMP (SAS Institute Inc.) software. All tests will be two-tailed and considered statistically significant when alpha level < 0.05.

6.0 TIMELINE

Recruitment and enrolment in objective #1 will be ongoing, subjects selected from the patient population of the lipid clinic at the CHUL hospital centre. Based on the clinical experience of Dr. Jean

Bergeron (co-PI) at this clinic, objective #1 should be complete within the first 2 years. Objective #2 will take place during years 2 and 3 of the proposal.

7.0 INVESTIGATORS

Dr Denis R. Joanisse, PhD (specialist in human muscle metabolism), nominated PI, for muscle biopsy procedures and histological and biochemical analyses. Dr Jean Bergeron, MD FRCPC (lipidologist), co-principal investigator, for patient recruitment, clinical evaluation (medical history and questionnaires), clinical laboratory measurements and protocol supervision. Dr Jérôme Frenette PT, PhD (physical therapist and specialist in muscle damage, inflammation and dysfunction), co-principal investigator, for muscle strength and endurance measurements. Collaborators (please see letters of collaboration) include Dr Jean Doré, MD (sports medicine, Laval University), for muscle biopsies and Dr David Hood, PhD (specialist in human mitochondrial biogenesis, York University), for muscle mitochondrial protein profiling. Clinicians (colleagues of Dr. Jean Bergeron) at the CHUL lipid clinic will also collaborate in patient recruitment. In addition to research and clinical staff involvement, a PhD student will be recruited as a principle collaborator for this work.

8.0 CLINICAL RELEVANCE AND PERSPECTIVES

This study has basic research and clinical relevance. It will be the first study to use state of the art technology to assess muscle function and tissue changes in patients suffering from statin-related myalgia. It is important to consider that, at this time, no clinical tools, other than chronologically compatible medical history reports, are available to objectively assess statin-induced myalgia. Our data on muscle function and tissue changes could serve to establish objective measures to assess the severity of statin-induced MRSE.

The strategy we are using in this project presents an alternative approach to medication side effect management, beyond simply replacing the offending drug or changing its dose. The use of statins in combination with vitamin D supplementation, should it prove successful in reducing or eliminating MRSE, will offer a new approach for the treatment of dyslipidemic patients, certainly increasing the proportion of these patients that attain their LDL-cholesterol goals and decreasing their risk of cardiovascular disease.

Obvious avenues of research follow from the proposed project. The extent and impact of MRSE in other populations, such as young adults, children (who are likely to become the target of statin prescription sooner than later), or athletes, has yet to be studied systematically. Should we succeed in demonstrating that vitamin supplementation can relieve myalgia in patients under statin therapy, studies into the optimization of this approach will be needed, as will examination in greater detail of the mechanisms that account for this effect.

Other future studies could attempt to identify a blood marker profile identifying patients at risk of, or suffering from, muscle damage due to statin use. Also, genetic profiling, focusing on mutations recently described and found to be associated with statin-induced myopathy [60, 61], could be related to any of the phenomena we identify in the present study and warrant future examination.
